# The orbitofrontal cortex: reward, emotion and depression

**DOI:** 10.1093/braincomms/fcaa196

**Published:** 2020-11-16

**Authors:** Edmund T Rolls, Wei Cheng, Jianfeng Feng

**Affiliations:** 1 Oxford Centre for Computational Neuroscience, Oxford, UK; 2 Department of Computer Science, University of Warwick, Coventry CV4 7AL, UK; 3 Institute of Science and Technology for Brain-inspired Intelligence, Fudan University, Shanghai 200433, China; 4 School of Mathematical Sciences, School of Life Science and the Collaborative Innovation Center for Brain Science, Fudan University, Shanghai 200433, China; 5 Key Laboratory of Computational Neuroscience and Brain-Inspired Intelligence, Fudan University, Ministry of Education, Shanghai 200433, China

**Keywords:** depression, orbitofrontal cortex, reward, emotion, decision-making

## Abstract

The orbitofrontal cortex in primates including humans is the key brain area in emotion, and in the representation of reward value and in non-reward, that is not obtaining an expected reward. Cortical processing before the orbitofrontal cortex is about the identity of stimuli, i.e. ‘what’ is present, and not about reward value. There is evidence that this holds for taste, visual, somatosensory and olfactory stimuli. The human medial orbitofrontal cortex represents many different types of reward, and the lateral orbitofrontal cortex represents non-reward and punishment. Not obtaining an expected reward can lead to sadness, and feeling depressed. The concept is advanced that an important brain region in depression is the orbitofrontal cortex, with depression related to over-responsiveness and over-connectedness of the non-reward-related lateral orbitofrontal cortex, and to under-responsiveness and under-connectivity of the reward-related medial orbitofrontal cortex. Evidence from large-scale voxel-level studies and supported by an activation study is described that provides support for this hypothesis. Increased functional connectivity of the lateral orbitofrontal cortex with brain areas that include the precuneus, posterior cingulate cortex and angular gyrus is found in patients with depression and is reduced towards the levels in controls when treated with medication. Decreased functional connectivity of the medial orbitofrontal cortex with medial temporal lobe areas involved in memory is found in patients with depression. Some treatments for depression may act by reducing activity or connectivity of the lateral orbitofrontal cortex. New treatments that increase the activity or connectivity of the medial orbitofrontal cortex may be useful for depression. These concepts, and that of increased activity in non-reward attractor networks, have potential for advancing our understanding and treatment of depression. The focus is on the orbitofrontal cortex in primates including humans, because of differences of operation of the orbitofrontal cortex, and indeed of reward systems, in rodents. Finally, the hypothesis is developed that the orbitofrontal cortex has a special role in emotion and decision-making in part because as a cortical area it can implement attractor networks useful in maintaining reward and emotional states online, and in decision-making.

## Teaser

The orbitofrontal cortex is involved in emotion, reward value and reward-related decision-making. Rolls *et al.* describe how it implements these functions, and the effects of damage. In depression, the lateral orbitofrontal cortex has increased functional connectivity and sensitivity to non-reward; and the medial orbitofrontal cortex has decreased connectivity and sensitivity to reward.

## Graphical Abstract

**Graphical Abstract:**
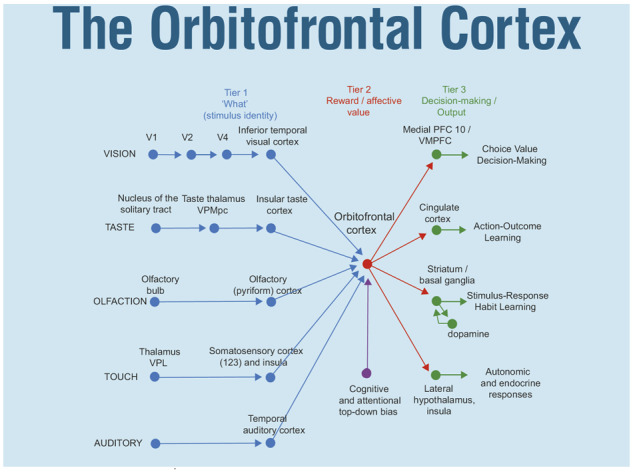

## Introduction

**Figure 1:**
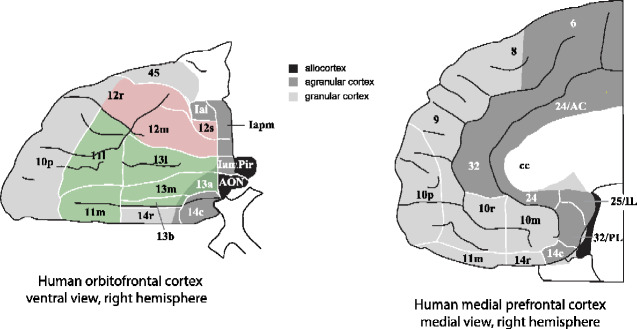
**Maps of architectonic areas in the orbitofrontal cortex and medial prefrontal cortex of humans.** Left, ventral view of the brain: The medial OFC includes areas 13 and 11 (green). The lateral OFC includes area 12 (red). (Area 12 is sometimes termed area 12/47 in humans. This figure shows two architectonic subdivisions of area 12.) Almost all of the human OFC except area 13a is granular. Agranular cortex is shown in dark grey. The part of area 45 shown is the orbital part of the inferior frontal gyrus pars triangularis. Right: the anterior cingulate cortex (medial view) includes the parts shown of areas 32, 25 (subgenual cingulate) and 24. The ventromedial prefrontal cortex includes areas 14 (gyrus rectus) 10 m and 10r. AON—anterior olfactory nucleus; Iai, Ial, Iam, Iapm—subdivisions of the agranular insular cortex [after Öngür *et al.* (2003)*Journal of Comparative Neurology* with permission of John Wiley & Sons, Inc., modified from a redrawn version by Passingham and Wise (2012).].

Advances in our understanding of the orbitofrontal cortex (OFC) are described here, and include the following. First, the OFC includes the medial OFC areas 13 and 11, and the lateral OFC area 12, as shown in Fig. 1. Conclusions about the functions of the OFC cannot be established by considering only one part of it, the medial OFC areas 13 and 11 (Rudebeck *et al.*, 2013). Furthermore, some authors have termed a region that includes the lateral OFC area 12 the ‘ventrolateral prefrontal cortex’ (Rudebeck *et al.*, 2017), and that terminology has clouded the issue in such studies of the functions of the lateral OFC, area 12. Dividing the OFC into a medial part (areas 13 and 11) and a lateral part (area 12) (based on its architectonics, Fig. 1) is useful, for connectivity-based parcellations show differences in the connectivity of these parts in humans (Hsu *et al.*, 2020; Du *et al.*, 2020a).

A second key point made is that this distinction between the human medial and lateral OFC is important functionally: the lateral OFC (area 12, Fig. 1) is implicated in the effects of aversive and subjectively unpleasant stimuli, and in not receiving expected rewards (termed ‘non-reward’) when a reward choice must be reversed and has increased functional connectivity in depression. In contrast, the medial OFC (areas 13 and 11, Fig. 1) is activated by rewarding and subjectively pleasant stimuli and has reduced functional connectivity in depression. This difference is supported by recent investigations in, for example, a monetary Win versus NoWin task (Xie *et al.*, 2020).

A third key advance is in understanding the connectivity of the human OFC using both tractography (Hsu *et al.*, 2020) and functional connectivity (Du *et al.*, 2020a), showing, for example, that the medial OFC is connected especially with the reward-related pregenual anterior cingulate cortex, and the lateral OFC and its closely related orbitofrontal part of the inferior frontal gyrus is connected especially with the non-reward and punishment-related supracallosal part of the anterior cingulate cortex. This is relevant to increasing understanding that the orbitofrontal is a key brain region involved in reward value and its rapid updating in even one trial when the reward value changes, and sends this information to the anterior cingulate cortex for actions to be learned guided by the reward or non-reward outcomes received (Rolls, 2019b).

A fourth key advance included here is that the anterior cingulate cortex can be conceptualized as receiving information about reward outcomes from the OFC, and the posterior cingulate cortex as receiving information from the parietal cortex about actions just performed, providing the signals needed for action–outcome goal-related learning, and sending outputs to premotor areas from the mid-cingulate cortex (Rolls, 2019b).

A fifth set of key advances described here is in understanding the functions of the OFC in mental disorders including depression by using voxel-level analyses of functional connectivity in many recent large-scale studies. Indeed, it is a key aim of this article to highlight the potential importance of the OFC, as a key brain region involved in emotion, in understanding and treating depression.

A sixth key feature of this article is the increasing evidence that the human and non-human primate OFC, with its importance for reward representations and very rapid updating of these that is important to social behaviour is very different from the rodent OFC, for the rodent OFC, and the whole organization of reward processing in the rodent brain, is very different (Rolls, 2019c, 2021a). Partly for this reason, a feature of this article is that it focusses on the evidence from both humans and foundational studies in non-human primates, because of similarities in their OFC systems, and their differences from the processing in rodents.

This article refers to some of the key concepts developed in *The Orbitofrontal Cortex* (Rolls, 2019c), and has a different focus to an earlier article published in 2017 (Rolls, 2019d).

We start by describing new evidence on the connections of the OFC and its functions in reward and emotion and then consider new evidence on how differences in OFC function are related to depression.

## The orbitofrontal cortex is a key brain region in reward value, mood and emotion

### The connections of the orbitofrontal cortex

The OFC cytoarchitectonic areas of the human brain are shown in Fig. 1 (left). The medial OFC includes areas 13 and 11 (Öngür *et al.*, 2003). The lateral OFC includes area 12 (sometimes in humans termed 12/47). The anterior cingulate cortex includes the parts shown in Fig. 1 (right) of areas 32, 25 (subgenual cingulate) and 24. The ventromedial prefrontal cortex includes areas 14 (gyrus rectus), 10m and 10r.

**Figure 2:**
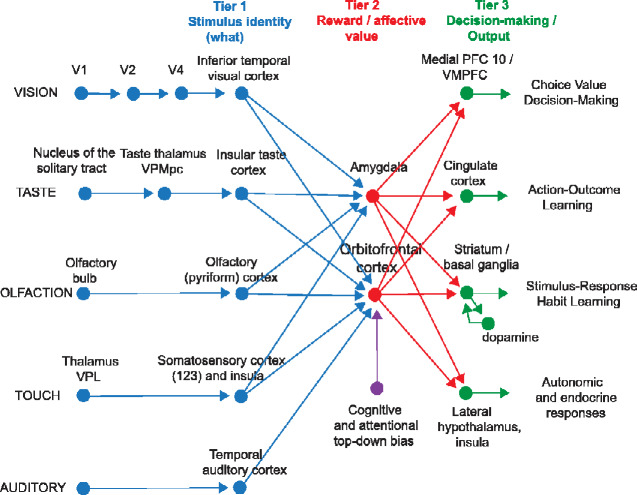
**Some of the connections of the taste, olfactory, somatosensory, visual and auditory pathways to the OFC and amygdala in primates.** V1, primary visual (striate) cortex; V2 and V4, further cortical visual areas. PFC, prefrontal cortex. The Medial PFC area 10 is part of the VMPFC. Ventro-postero-lateral (VPL) nucleus of the thalamus, which conveys somatosensory information to the primary somatosensory cortex (areas 1, 2 and 3). Ventro-postero-medial nucleus pars parvocellularis (VPMpc) of the thalamus, which conveys taste information to the primary taste cortex. For the purposes of description, the stages can be described as Tier 1, representing what object is present independently of reward value; Tier 2 in which reward value and emotion is represented; and Tier 3 in which decisions between stimuli of different value are taken, and in which value is interfaced to behavioural output systems. A pathway for top-down attentional and cognitive modulation of emotion is shown in purple. Auditory inputs also reach the amygdala (From Rolls, 2019c).

Some of the main connections of the OFC in primates are shown schematically in Fig. 2 (Carmichael and Price, 1994, 1995; Barbas, 1995; Petrides and Pandya, 1995; Pandya and Yeterian, 1996; Ongür and Price, 2000; Price, 2006, 2007; Barbas, 2007; Saleem *et al.*, 2008; Mackey and Petrides, 2010; Petrides *et al.*, 2012; Saleem *et al.*, 2014; Henssen *et al.*, 2016; Rolls, 2017, 2019c,d). The OFC receives inputs from the ends of the ventral cortical streams that process the identity of visual, taste, olfactory, somatosensory and auditory stimuli (Rolls, 2019c). At the ends of each of these cortical processing streams, the identity of the stimulus is represented independently of its reward value. This is shown by neuronal recordings in primates (Rolls, 2019c). For example, the inferior temporal cortex represents objects and faces independently of their reward value as shown by visual discrimination reversal and devaluation of reward tests by feeding to satiety (Rolls *et al.*, 1977; Rolls, 2012a, 2016b, 2019c). Similarly, the insular primary taste cortex represents what the taste is independently of its reward value (Yaxley *et al.*, 1988; Rolls, 2015, 2016c, 2019c).

Outputs of the OFC reach the anterior cingulate cortex (Rolls, 2019c), the striatum, the insula and the inferior frontal gyrus, and enable the reward value representations in the OFC to influence behaviour (Fig. 2, green). The OFC projects reward value information to the anterior cingulate cortex, where it is used to provide the reward outcomes for action–outcome learning (Rushworth *et al.*, 2012; Rolls, 2019a,c). The OFC projects reward-related information to the ventral striatum (Williams *et al.*, 1993), and this provides a route, in part via the habenula, for reward-related information to reach the dopamine neurons (Rolls, 2017), which respond inter alia to positive reward prediction error (Bromberg-Martin *et al.*, 2010; Schultz, 2016a, 2017). The striatal/basal ganglia route is used for stimulus–response, habit, learning (Everitt and Robbins, 2013; Rolls, 2014), with dopamine used to provide reward prediction error in reinforcement learning (Schultz, 2016b; Cox and Witten, 2019). As that system uses dopamine in reinforcement learning of stimulus–response habits, it is much less fast to learn than the OFC (reward or punishment outcome) with anterior cingulate cortex (action) system for action–outcome goal-based learning, and for emotion (Rolls, 2021a). The OFC may also have direct connections to the ventral tegmental area in mice where dopamine neurons are located (Namboodiri *et al.*, 2019). The OFC outputs to the insula include a projection to the viscero-autonomic cortex in the antero-ventral insula (Hassanpour *et al.*, 2018) that helps to account for the reason why the insula is activated in some tasks in which the OFC is involved (Rolls, 2016c, 2019c). The lateral OFC also projects to the inferior frontal gyrus (Hsu *et al.*, 2020; Du *et al.*, 2020a), to a region that on the right is implicated in stopping behaviour (Aron *et al.*, 2014), and to a region on the left that includes Broca’s area (Hsu *et al.*, 2020; Rolls, 2021a).

**Figure 3:**
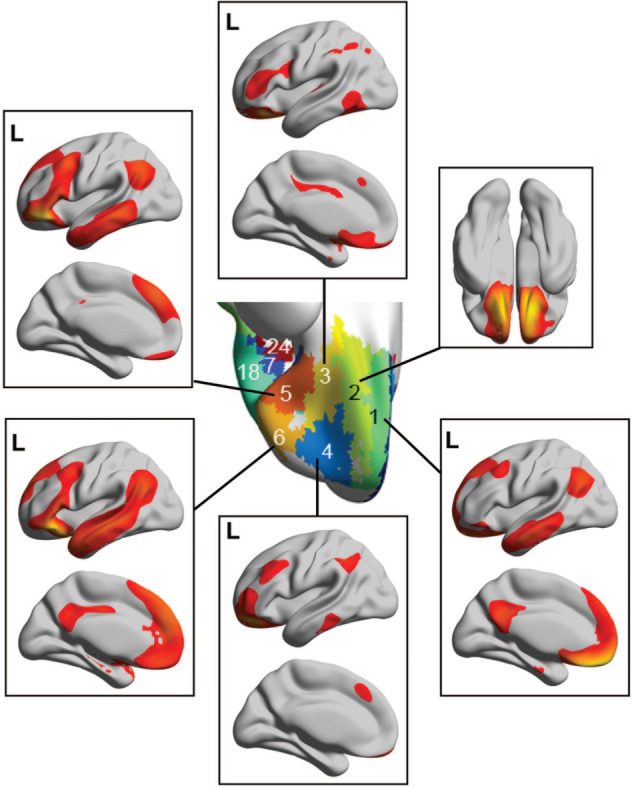
**Connectivity shown on surface maps of the brain of the different parcels or subdivisions of the human orbitofrontal cortex (OFC).** The parcels were based on the functional connectivity of every OFC voxel with each of the 94 automated anatomical labelling atlas 2 brain regions. Six divisions of the OFC are shown, with the approximate correspondence of each division with the cytoarchitectonic areas defined by Öngür *et al.* (2003) as shown in Fig. 1 as follows: 1—the gyrus rectus (much of it area 14); 2—medial OFC (area 13 m); 3—posterior OFC (area 13 l); 4—anterior OFC (area 11 l); 5—lateral OFC, posterior (area 12 m); 6—lateral OFC, anterior (area 12r). Surface maps showing the cortical connectivity of each parcel are shown. The functional connectivities have been thresholded at 0.3, and were obtained in resting-state fMRI with 654 participants. Quantitative evidence on the connectivity with different brain regions of each parcel is provided by Du *et al.* (2020b) and Hsu *et al.* (2020) (after Du *et al.*, 2020a).

New evidence on the connections of the OFC in humans is shown in Fig. 3, based on resting-state functional connectivity in 654 participants (Du *et al.*, 2020a). First, it is shown that a parcellation based on the voxel-wise functional connectivity of OFC voxels with other brain areas reveals sub-divisions (Fig. 3) that are very similar to the cytoarchitectural divisions of the human OFC shown in Fig. 1. Second, the lateral OFC (parcels 5 and 6, Fig. 3) has connectivity with language-related areas not only in the inferior frontal gyrus (Broca’s area) but also with the angular and supramarginal gyri. Parts of the medial OFC (parcels 2–4, Fig. 3) have connectivity with the parahippocampal gyrus, hippocampus, the temporal cortex and fusiform gyrus, the insula and the cingulate cortex. These connectivities, as shown below, are altered in opposite directions in depression.

The connectivity of the OFC analysed in humans with functional connectivity is likely to include trans-synaptic effects, but direct connections have been investigated with diffusion tractography imaging in 50 participants (Hsu *et al.*, 2020). The medial OFC and ventromedial prefrontal cortex have direct connections with the pregenual and subgenual parts of the anterior cingulate cortex, all of which are reward-related areas. The lateral OFC and its closely connected right inferior frontal gyrus have direct connections with the supracallosal anterior cingulate cortex, all of which are punishment or non-reward-related areas (Hsu *et al.*, 2020). This confirms the findings based on functional connectivity for connections between the medial OFC and the pregenual cingulate cortex; and the lateral OFC and related right inferior frontal gyrus with the supracallosal anterior cingulate cortex (Rolls *et al.*, 2019; Du *et al.*, 2020a). The lateral OFC and right inferior frontal gyrus also have direct connections with the right supramarginal gyrus and inferior parietal cortex, and with some premotor cortical areas, which may provide outputs for the lateral OFC and right inferior frontal gyrus. Direct connections of the human OFC and inferior frontal gyrus with the temporal lobe were especially with the temporal pole (Hsu *et al.*, 2020).

### The medial orbitofrontal cortex represents reward value; and the lateral orbitofrontal cortex represents punishers and non-reward

**Figure 4:**
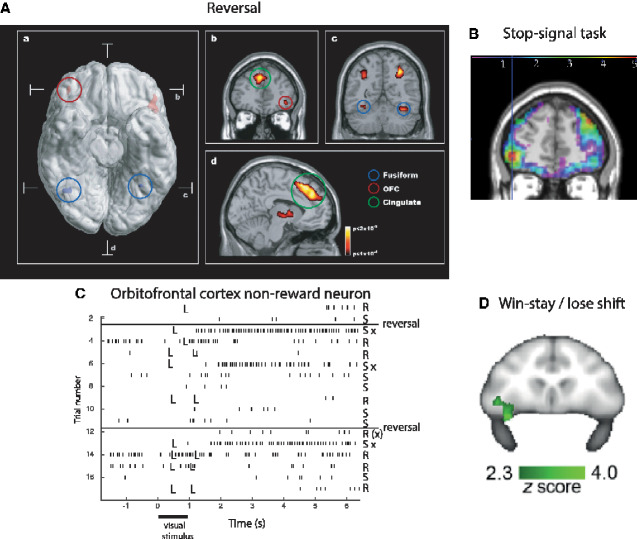
**Evidence that the human lateral OFC is activated by non-reward.** Activation of the lateral OFC in a visual discrimination reversal task on reversal trials, when a face was selected but the expected reward was not obtained, indicating that the subject should select the other face in future to obtain the reward. (**A**) A ventral view of the human brain with indication of the location of the two coronal slices (**A**, **C**) and the transverse slice (d). The activations with the red circle in the lateral OFC (peaks at [42 42 −8] and [−46 30 −8]) show the activation on reversal trials compared to the non-reversal trials. For comparison, the activations with the blue circle show the fusiform face area produced just by face expressions, not by reversal, which are also indicated in the coronal slice in **C**. (**B**) A coronal slice showing the activation in the right OFC on reversal trials. Activation is also shown in the supracallosal anterior cingulate region (Cingulate, green circle) that is also known to be activated by many punishing, unpleasant, stimuli (Grabenhorst and Rolls, 2011) (from Kringelbach and Rolls, 2003). (**B**) Activations in the human lateral OFC are related to a signal to change behaviour in the stop-signal task. In the task, a left or right arrow on a screen indicates which button to touch. However, on some trials, an up-arrow then appears, and the participant must change the behaviour and stop the response. There is a larger response on trials on which the participant successfully changes the behaviour and stops the response, as shown by the contrast stop–success—stop–failure, in the ventrolateral prefrontal cortex in a region including the lateral OFC, with peak at [−42 50 −2] indicated by the cross-hairs, measured in 1709 participants. There were corresponding effects in the right lateral OFC [42 52 −4]. Some activation in the dorsolateral prefrontal cortex in an area implicated in attention is also shown (after Deng *et al.*, 2017). (**C**) Non-reward error-related neurons maintain their firing after non-reward is obtained. Responses of an OFC neuron that responded only when the macaque licked to a visual stimulus during reversal, expecting to obtain fruit juice reward, but actually obtained the taste of aversive saline because it was the first trial of reversal (trials 3, 6 and 13). Each vertical line represents an action potential; each L indicates a lick response in the Go-NoGo visual discrimination task. The visual stimulus was shown at time 0 for 1 s. The neuron did not respond on most reward (R) or saline (S) trials, but did respond on the trials marked S x, which were the first or second trials after a reversal of the visual discrimination on which the monkey licked to obtain reward, but actually obtained saline because the task had been reversed. The two times at which the reward contingencies were reversed are indicated. After responding to non-reward, when the expected reward was not obtained, the neuron fired for many seconds, and was sometimes still firing at the start of the next trial. It is notable that after an expected reward was not obtained due to a reversal contingency being applied, on the very next trial the macaque selected the previously non-rewarded stimulus. This shows that rapid reversal can be performed by a non-associative process, and must be rule-based. (After Thorpe *et al.*, 1983). (**D**) BOLD signal in the macaque lateral orbitofrontal related to win-stay/lose-shift performance, that is, to reward reversal performance (after Chau *et al.*, 2015).

The primate including human OFC is the first stage of cortical processing that represents *reward value* (red, Fig. 2) (Rolls, 2019c). For example, in devaluation experiments, taste, olfactory, visual and oral texture neurons in the macaque orbitofrontal respond to food when hunger is present, and not after feeding to satiety when the food is no longer rewarding (Rolls *et al.*, 1989; Critchley and Rolls, 1996). In visual discrimination reversal experiments, neurons in the macaque OFC reverse the visual stimulus in as little as one trial when the reward versus punishment taste received as an outcome for the choice reverses (Thorpe *et al.*, 1983; Rolls *et al.*, 1996). This is rule-based reversal, in that after a previously rewarded visual stimulus is no longer rewarded, the macaques choose the other stimulus on the very next trial, although its previous reward association was with punishment, as shown in Fig. 4 which illustrates a non-reward neuron active at the time of the reversal (Thorpe *et al.*, 1983). (Non-reward refers here to not obtaining an expected reward.) This capability requires a rule to be held in memory and reversed by non-reward (Deco and Rolls, 2004; Rolls and Deco, 2016), is very appropriate for primates which in social situations may benefit from being very responsive to non-reward versus reward signals, and may not occur in rodents (Rolls, 2019c, 2021a; Hervig *et al.*, 2020). The human lateral OFC is activated in this one-trial rule-based non-associative reversal (Rolls *et al.*, 2020b). The macaque OFC contains neurons that reflect face expression and face identity (both necessary to decode value and important in social behaviour) (Thorpe *et al.*, 1983; Rolls *et al.*, 2006), and also social categories such as young faces (Barat *et al.*, 2018) and position in the social hierarchy (Munuera *et al.*, 2018). Economic value is represented in the OFC, in that, for example, single neurons reflect the trade-off between the quality of a reward and the amount that is available (Padoa-Schioppa and Conen, 2017). These investigations show that some OFC neurons respond to outcome value (e.g. the taste of food), and others to expected value (or future rewards), and interestingly humans with ADHD have increased sensitivity to these future rewards (Tegelbeckers *et al.*, 2018). The expected value neurons are not positive reward prediction error neurons, for they keep responding to the expected reward even when there is no prediction error (Rolls, 2021a). Consistent with this neurophysiological evidence, lesions of the macaque medial OFC areas 13 and 11 make the animals less sensitive to reward value, as tested in devaluation experiments in which the animal is fed to satiety (Rudebeck *et al.*, 2017). (These were described as OFC lesions, but in fact, included primarily the medial OFC areas 13 and 11 shown in Fig. 1.)

**Figure 5:**
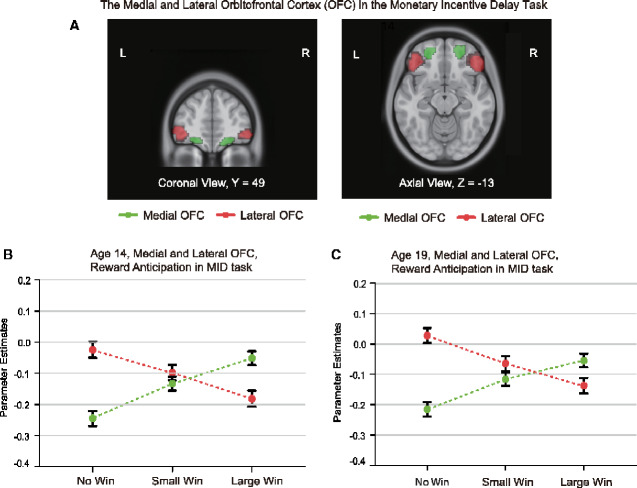
**The lateral OFC is activated by not winning, and the medial OFC by winning, in the monetary incentive delay task.** The lateral OFC region in which activations increased towards no reward (No Win) in the monetary incentive delay task are shown in red in 1140 participants at age 19 and in 1877 overlapping participants at age 14. The conditions were Large Win (10 points) to Small Win (2 points) to No Win (0 points) (at 19; sweets were used at 14). The medial OFC region in which activations increased with increasing reward from No Win to Small Win to High Win) is shown in green. The parameter estimates are shown from the activations for the participants (mean ± sem) with the lateral orbitofrontal in red and medial OFC in green. The interaction term showing the sensitivity of the medial OFC to reward and the lateral OFC to non-reward was significant at *P* = 10^−50^ at age 19 and *P* < 10^−72^ at age 14. In a subgroup with depressive symptoms as shown by the Adolescent Depression Rating Scale, it was further found that there was a greater activation to the No Win condition in the lateral OFC; and the medial OFC was less sensitive to the differences in reward value (modified from Xie *et al.*, 2020).

Neuroimaging experiments in humans (‘Technical Note’) produce consistent evidence about *reward value* representations (de Araujo *et al.*, 2003; Kringelbach and Rolls, 2003; Kringelbach *et al.*, 2003; Grabenhorst and Rolls, 2008; Grabenhorst *et al.*, 2008a), and allow the types of reward to be extended to include monetary reward (O’Doherty *et al.*, 2001; Xie *et al.*, 2020), face expressions (Kringelbach and Rolls, 2003) and face beauty (O’Doherty *et al.*, 2003). In humans, the medial OFC is activated by many rewarding stimuli and reflects their subjective pleasantness (Grabenhorst and Rolls, 2011; Rolls, 2019c). This is found for odours (Rolls *et al.*, 2003a), flavour (de Araujo *et al.*, 2003; Kringelbach *et al.*, 2003), pleasant touch (Rolls *et al.*, 2003b; McCabe *et al.*, 2008), monetary reward (O’Doherty *et al.*, 2001; Xie *et al.*, 2020) and amphetamine (Völlm *et al.*, 2004). A recent study with 1877 participants emphasizes these points, by showing that the medial OFC is activated by reward (such as winning money or candies) and that the lateral OFC is activated by not winning (Fig. 5) (Xie *et al.*, 2020). Humans with OFC lesions may also be less sensitive to reward, as shown by their reduced subjective emotional feelings and altered social and emotional behaviour and problems with face and voice expression processing (Rolls *et al.*, 1994; Hornak *et al.*, 1996, 2003; Rolls, 2021b).

The macaque OFC has neurons that respond when an expected reward is not received (Thorpe *et al.*, 1983), and these have been termed *non-reward neurons* (Rolls, 2014, 2019c) (see example in Fig. 4C). They can be described as negative reward prediction error neurons, in that they respond when a reward outcome is less than was expected (Rolls, 2019c). These neurons do not respond to expected punishers [e.g. the discriminative stimulus for saline in Fig. 4C; (Thorpe *et al.*, 1983)], but other neurons do respond to expected punishers (Rolls *et al.*, 1996), showing that non-reward and punishment are represented by different neurons in the OFC. The finding of non-reward neurons is robust, in that 18/494 (3.6%) of the neurons in the original study responded to non-reward (Thorpe *et al.*, 1983), consistent results were found in different tasks in a complementary study (10/140 non-reward neurons in the OFC or 7.1%) (Rosenkilde *et al.*, 1981), and an fMRI study has shown that the macaque lateral OFC is activated when an expected reward is not obtained during reversal (Chau *et al.*, 2015) (Fig. 4D). The hypothesis is that these non-reward neurons are computed in the OFC, because this is the first brain region in primates at which expected value and outcome value are represented, and these two signals are those required to compute non-reward, that is, reward outcome < expected value (as shown in Fig. 2) and with the evidence set out fully by Rolls (2019c, 2021a). Corresponding to this, the human lateral OFC is activated when a reward is not obtained in a visual discrimination reversal task (Kringelbach and Rolls, 2003) (Fig. 4A), and when money is not received in a monetary reward task (O’Doherty *et al.*, 2001; Xie *et al.*, 2020), and in a one-trial rule-based reward reversal task (Rolls *et al.*, 2020b). Consistent with this, the human lateral OFC is also activated by punishing, subjectively unpleasant, stimuli (Grabenhorst and Rolls, 2011; Rolls, 2019c). Examples include unpleasant odours (Rolls *et al.*, 2003a), pain (Rolls *et al.*, 2003b), losing money (O’Doherty *et al.*, 2001) and receiving an angry face expression, indicating that behaviour should change in a reversal (Kringelbach and Rolls, 2003). The human right lateral OFC/inferior frontal gyrus is also activated when behavioural correction is required in the stop-signal task (Fig. 4B) (Deng *et al.*, 2017). These discoveries show that one way in which the OFC is involved in decision-making is by representing rewards, punishers and errors made during decision-making. This is supported by the problems that OFC damage produces in decision-making, which include failing to respond correctly to non-reward, as described next.

Consistent with this neurophysiological and neuroimaging evidence, lesions of the OFC can impair behavioural changes to *non-reward*. For example, reward reversal learning is impaired during decision-making in humans, who continue responding to the previously rewarded, now non-rewarded, stimulus (Rolls *et al.*, 1994; Hornak *et al.*, 2004; Fellows, 2011). The change in contingency between the stimulus and the reward versus non-reward is not processed correctly. In macaques, damage to the lateral OFC impairs reversal and extinction (Butter, 1969; Iversen and Mishkin, 1970). It has been a problem in some studies of the orbitofrontal cortex in macaques that the OFC lesions have been incomplete, with for, example, the lesions including only the medial areas 13 and 11, and not the lateral OFC area 12, with one study reporting no reversal learning deficit after such a medial lesion, and suggesting that the OFC was not involved in reversal (Rudebeck *et al.*, 2013). In the light of the above, that does not address the role of the OFC in reversal learning, as including the lateral OFC area 12 is highly relevant. In a more recent study, damage to the lateral OFC (mainly area 12 as shown in Fig. 1, and extending around the inferior convexity, but described as VLPFC—ventrolateral prefrontal cortex) was found to impair the ability to make choices based on whether reward versus non-reward had been received (Rudebeck *et al.*, 2017; Murray and Rudebeck, 2018), which is the type of contingency learning in which this brain region is implicated (Rolls, 2019c; Rolls *et al.*, 2020b). (Unfortunately, the one-trial, rule based, reversal learning in which the OFC is implicated (Rolls, 2019c), was not tested in that study (Rudebeck *et al.*, 2017).) Further evidence that the lateral OFC is involved in learning contingencies between stimuli and reward versus non-reward is that in humans, lateral OFC damage impaired this type of ‘credit assignment’ (Noonan *et al.*, 2017). This type of flexibility of behaviour is important in primate including human social interactions (Rolls, 2018a, 2019c).

### The ventromedial prefrontal cortex and reward-related decision-making

The ventromedial prefrontal cortex (VMPFC, which can be taken to include the gyrus rectus area 14 and parts of 10m and 10r, Fig. 1) receives inputs from the OFC and has distinct connectivity [with strong functional connectivity with the superior medial prefrontal cortex, cingulate cortex and angular gyrus (Du *et al.*, 2020a)]. The VMPFC has long been implicated in reward-related decision-making (Bechara *et al.*, 1997, 2005; Glascher *et al.*, 2012), this region is activated during decision-making contrasted with reward valuation (Rolls and Grabenhorst, 2008; Grabenhorst *et al.*, 2008b), and it has the signature of a decision-making region of increasing its activation in proportion to the difference in the decision variables, which correlates with decision confidence (Rolls *et al.*, 2010a,b; Rolls, 2019c). Consistently, single neurons in the macaque ventromedial prefrontal cortex signal the value of the chosen offer, suggesting that the network produces a choice (Strait *et al.*, 2014), also consistent with the attractor model of decision-making (Rolls *et al.*, 2010a,b; Rolls, 2014, 2016b, 2021a). The attractor model of decision-making is a neuronal network with associatively modifiable recurrent collateral synapses between the neurons of the type prototypical of the cerebral cortex (Wang, 2002; Rolls and Deco, 2010; Rolls, 2021a). The decision variables are applied simultaneously, and the network, after previous training with these decision variables, reaches a state where the population of neurons representing one of the decision variables has a high firing rate (Rolls and Deco, 2010; Deco *et al.*, 2013; Rolls, 2016b; 2021a).

### The orbitofrontal cortex and emotion

**Figure 6:**
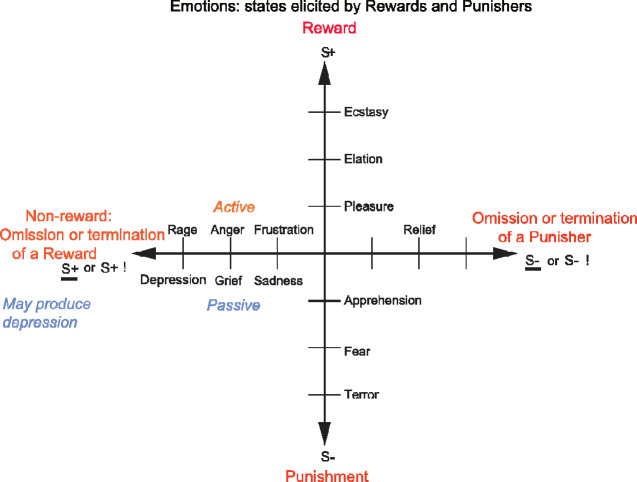
**Some of the emotions associated with different reinforcement contingencies.** Intensity increases away from the centre of the diagram, on a continuous scale. The classification scheme created by the different reinforcement contingencies consists with respect to the action of (1) the delivery of a reward (S+), (2) the delivery of a punisher (S−), (3) the omission of a reward (S−) (extinction) or the termination of a reward (S+!) (time out) and (4) the omission of a punisher (S−) (avoidance) or the termination of a punisher (S−!) (escape). It is noted that the vertical axis describes emotions associated with the delivery of a reward (up) or punisher (down). The horizontal axis describes emotions associated with the non-delivery of an expected reward (left) or the non-delivery of an expected punisher (right). For the contingency of non-reward (horizontal axis, left), different emotions can arise depending on whether an active action is possible to respond to the non-reward, or whether no action is possible, which is labelled as the passive condition. In the passive condition, non-reward may produce depression. The diagram summarizes emotions that might result for one reinforcer as a result of different contingencies. Every separate reinforcer has the potential to operate according to contingencies such as these. This diagram does not imply a dimensional theory of emotion, but shows the types of emotional state that might be produced by a specific reinforcer. Each different reinforcer will produce different emotional states, but the contingencies will operate as shown to produce different specific emotional states for each different reinforcer.

One of the major theories of emotion is that emotions are states elicited by rewards and punishers, which are instrumental reinforcers (Rolls, 2000, 2013b, 2014, 2018a) (Fig. 6). The evidence described above shows that the OFC is involved in representing the reward value of stimuli (with an emphasis on the medial and mid-OFC areas 11 and 13); and is involved in learning associations between stimuli and rewards, and rapidly correcting these (with an emphasis on the lateral OFC area 12 and the closely connected orbital part of the inferior frontal gyrus) (Rolls, 2018a, 2019c). In this context, the theory of emotion holds that the role of the OFC in emotion is to decode the reward/punishment goals for action, by representing reward value, and by learning about stimuli with reward versus non-reward contingencies, and then to transmit the resulting representations to further brain regions (such as the cingulate cortex) which implement the learning of actions to obtain the reward outcomes signalled by the OFC (Rolls, 2019a,b; Rolls, 2021a). In accordance with this, the rewarding and punishing stimuli described above are all affective stimuli, and activate the OFC; and OFC damage impairs subjective emotional states (Hornak *et al.*, 2003, Rolls, 2019c), emotional responses to stimuli such as face and voice expression (Hornak *et al.*, 1996, 2003), and emotional and social behaviour, with the neurological evidence described in more detail elsewhere (Rolls, 2019c, 2021b). Furthermore, activations in the OFC are linearly related to the subjective pleasantness (i.e. affective experience) of stimuli, as described above, and elsewhere in more detail (Rolls, 2019c). The brain bases of subjective experience are a topic of considerable current interest, not only with higher order thought theories (*Rosenthal, 2004*; Brown *et al.*, 2019) but also with the higher order syntactic thought theory of consciousness (Rolls, 2007, 2012b, 2014, 2016b, 2018a, 2020) which is more computationally specific and addresses the adaptive value of the type of processing related to consciousness. The point made here is that the OFC is at least on the route to human subjective experience of emotion and affective value (Rolls, 2019c).

Although the amygdala has many of the same connections as the OFC (Fig. 2), it is an evolutionarily old brain region, and appears to be overshadowed by the OFC in humans, in that the effects of damage to the human amygdala on emotion and emotional experience are much more subtle (Whalen and Phelps, 2009; Delgado *et al.*, 2011; LeDoux and Pine, 2016; LeDoux *et al.*, 2018) than that of damage to the OFC (Rolls *et al.*, 1994; Hornak *et al.*, 1996, 2003, 2004; Camille *et al.*, 2011; Fellows, 2011; Rolls, 2019c). Indeed, LeDoux and colleagues have emphasized the evidence that the human amygdala is rather little involved in subjective emotional experience (LeDoux and Pine, 2016; LeDoux and Brown, 2017; LeDoux *et al.*, 2018). That is in strong contrast to the OFC, which is involved in subjective emotional experience, as shown by the evidence just cited. The OFC provides the answer to LeDoux’s conundrum: if not the amygdala for subjective emotional experience, then what? Furthermore, consistent with the poor rapid reversal learning found by amygdala neurons (Sanghera *et al.*, 1979; Rolls, 2014) compared to OFC neurons, it has been found that neuronal responses to reinforcement predictive cues in classical conditioning update more rapidly in the macaque OFC than amygdala, and activity in the OFC but not the amygdala was modulated by recent reward history (Saez *et al.*, 2017). Neurons that are sensitive to the rank of the individual being viewed in the social hierarchy are found not only in the macaque amygdala, but also in the closely connected OFC, and anterior cingulate cortex (Munuera *et al.*, 2018). In addition, it has been shown in the macaque that amygdala neurons are involved in social, observational learning in a reversal-learning task, and that some neurons even predicted the choices of the partner monkey (Grabenhorst *et al.*, 2019). These processes—assessing the social rank of individuals, learning from social partners, anticipating their behaviour—are critical for social life. However, the balance may shift towards the OFC in humans, in that it is OFC damage in humans that produces profound changes in social and emotional behaviour, and subjective emotional experience, as well as in reward reversal learning (Rolls *et al.*, 1994; Hornak *et al.*, 1996; 2003; Berlin *et al.*, 2004, 2005; Hornak *et al.*, 2004; Rolls, 2019c).

### The rodent orbitofrontal cortex and reward systems

The focus in this article is on evidence from primates including humans. The reason for this focus is that the rodent OFC (Schoenbaum *et al.*, 2009; Wilson *et al.*, 2014; Sharpe *et al.*, 2015; Izquierdo, 2017; Sharpe *et al.*, 2019), and the whole operation of reward systems in rodents, appears to be somewhat different from that in primates including humans (Rolls, 2019c, 2021a), as follows.

First, the rodent OFC contains only agranular cortex, which corresponds to only a small region of the primate OFC, posteriorly (Wise, 2008; Passingham and Wise, 2012; Passingham, 2021), and these authors provide evidence that there is no equivalent in rodents of most of the primate OFC.

Second, the connectivity of the rodent reward systems including the OFC is so different from that of primates that the principles of operation appear to be very different. One example is the taste system, which in primates proceeds mainly via thalamo-cortical processing through a primary taste cortex in the insula to the OFC, whereas instead, rodents have a pontine taste area which projects taste information to many subcortical areas (Scott and Small, 2009; Small and Scott, 2009; Rolls, 2015, 2016c, 2019c). A second example is that with the great development of the temporal lobe in primates, visual processing becomes highly elaborated and transmits information about face identity and face expression to the OFC, where it can be used in emotional and social behaviour appropriate for different individuals, given the face expression and gestures (including face view) of each individual (Rolls, 2016c; 2019c). A third example is that because the visual representation in primates includes processing to the level of view-invariant representations of objects and faces, reward value-related learning in the OFC is efficient, for after a value association is made to one view, it generalizes to other views or transforms (Rolls, 2012a, 2016c, 2021a).

Third, in rodents, reward value as indicated in devaluation (satiety) studies involves reward processing even far peripherally in the first central relay, the nucleus of the solitary tract for taste (Giza and Scott, 1983, 1987; Giza *et al.*, 1992), and the olfactory bulb for odour (Pager *et al.*, 1972), making these complex systems, as reward and identity processing about taste and odour are entangled throughout the system with each other. In another example, in reward reversal in mice, the OFC has reward-related top-down effects on the primary somatosensory cortex (Banerjee *et al.*, 2020). This makes reward processing in rodents difficult to analyse. In contrast, in primates and humans, there is a clear separation between perceptual representations (Tier 1, Fig. 2), and reward value representations in the OFC and amygdala (Tier 2, Fig. 2) (Rolls, 2015, 2016b, 2019c, 2021a).

Fourth, although reward value is represented in the rodent (agranular) OFC, so also apparently are behavioural responses (Schoenbaum *et al.*, 2009; Wilson *et al.*, 2014; Sharpe *et al.*, 2015, 2019; Izquierdo, 2017), which makes the rodent OFC very different to the primate OFC. The primate OFC, in contrast, appears to specialize in reward (and of course punishment and non-reward) value representations, but not in interfacing these value representations to actions [which occurs in the primate cingulate cortex ([Bibr fcaa196-B159][Bibr fcaa196-B163])] or to responses (which occurs in the striatum and other parts of the basal ganglia), for actions and responses are poorly if at all represented in the primate OFC (Thorpe *et al.*, 1983; Padoa-Schioppa and Assad, 2006; Grattan and Glimcher, 2014; Rolls, 2019c). In contrast, in rodents, the OFC seems much more heterogeneous with behavioural responses also represented in it (Wilson *et al.*, 2014; Sharpe *et al.*, 2015, 2019), and again, the system is more complex because different computations are apparently intermingled in the same brain region. Although it is interesting that in the mouse, neurons in LO represent the values of individual options, the binary choice outcome and the chosen value, this is in the context of spatial responses (Kuwabara *et al.*, 2020), not of the value of goods as in primates.

Fifth, although reward reversal learning is studied in rodents (Hervig *et al.*, 2020), it does not so far appear to be of the same powerful type as the rule-based system present in primates, which allows switching to a different stimulus even previously associated with punishment when no reward is received when it was expected by a behavioural choice on a single trial (Thorpe *et al.*, 1983; Rolls *et al.*, 1996, 2020b; Rolls, 2019c). This type of rapid, rule-based, reversal provides a foundation for rapid changes in social behaviour whenever feedback is received, and a similar rule-based system is not known to be present in rodents. This is consistent with the great development of cortical processing for these functions provided by the primate OFC, given that the cortex provides a computational basis in its attractor networks for holding information online, and therefore producing behaviour that depends on ‘hidden’ internal states, rather than being more dominated by sensory input (Rolls, 2016b, 2021a). However, it is of interest that neurons in the rodent lateral OFC respond in reward reversal and that silencing these neurons impairs the reversal (Banerjee *et al.*, 2020).

Because we wish the advances described here to be relevant to understanding the functions of the OFC in humans, we focus here on the findings in primates including humans, but further evidence on research in rodents is provided elsewhere (Izquierdo, 2017; Rolls, 2019c, 2021a).

## A theory of depression

Better understanding of the functions of the OFC in major depressive disorder is important, for it is ranked by the World Health Organization as the leading cause of years-of-life lived with disability (Drevets, 2007; Gotlib and Hammen, 2009; Hamilton *et al.*, 2013). Moreover, the economic cost of depression is enormous, with an estimated 350 million people affected globally. For example, the cost to Europe of work-related depression was estimated to be Euro 617 billion annually in 2013 and rising (Matrix, 2013).

A theory of depression has been developed based on our understanding of the brain processes involved in emotion, reward and non-reward described above (Rolls, 2016a, 2018a). Given that not receiving expected rewards is a reinforcement contingency that can lead to sadness, or in the extreme case such as the loss of a loved one, depression, the theory was proposed that the lateral OFC, implicated in non-reward and learning contingencies between stimuli and reward versus non-reward, over-responds to non-reward in people with depression; and that a major non-reward event that activated the lateral OFC might lead to depression (Rolls, 2016a, 2018a). Because non-reward neurons in the lateral OFC can maintain their activity for at least many seconds (Fig. 4) (Thorpe *et al.*, 1983), and because this persistent activity is needed to ensure that after non-reward, the behaviour changes even if the same stimuli are not received for some time, the theory is that there is a non-reward attractor network in the lateral OFC, and that this is more sensitive or persistent in depression (Rolls, 2016a, 2018a). It is postulated that the effects of the non-reward can be prolonged by rumination of sad thoughts which is supported by a long loop attractor involving language areas in the angular gyrus and related regions, which receive inputs from the lateral OFC, and project back to it. The theory thus is that some aspects of depression may be related to over-responsiveness of the lateral orbitofrontal to non-reward and punishment (Rolls, 2016a, 2018a, 2021a). Consistent with this, increased sensitivity to non-reward (not winning in a monetary incentive delay task) of the lateral OFC is associated with the severity of depressive symptoms measured in hundreds of adolescents (Xie *et al.*, 2020).

Given that the activations of the lateral and medial OFC often appear to be reciprocally related (O’Doherty *et al.*, 2001; Rolls *et al.*, 2003a; Xie *et al.*, 2020), the other part of the theory is that in depression there may be underactivity, under-sensitivity or under-connectivity of the (reward-related) medial OFC in depression (Rolls, 2016a, 2018a). The theory is further that under-responsiveness of the medial OFC could contribute to other aspects of depression, such as anhedonia.

There is now much evidence that supports this theory of depression, as described in the following sections. This approach based on advances in understanding the functions of the primate including human OFC also provides an approach to understanding how other brain systems such as the subgenual/subcallosal anterior cingulate cortex are implicated in depression (Mayberg *et al.*, 2016), in that they receive inputs from the OFC. This approach also adds to previous approaches relating to reward and punishment systems (Eshel and Roiser, 2010; McCabe *et al.*, 2012; Satterthwaite *et al.*, 2015), by being based on the theory of emotion described above and on the understanding of brain systems involved in emotion in primates including humans described above. Other contributing factors to depression are considered elsewhere (Rolls, 2018a).

## Increased functional connectivity of the non-reward related lateral orbitofrontal cortex, and decreased functional connectivity of the reward-related medial orbitofrontal cortex, in depression

This section describes many new large-scale voxel-level analyses of differences in the connectivity of the OFC in depression, and this is the first review in which this new evidence is brought together. An overview of many of the new discoveries is shown in Fig. 8.

There is considerable interest in functional connectivity differences between people with major depressive disorder and controls, for increased functional connectivity could reflect increased communication between brain regions, and decreased connectivity the opposite. Functional connectivity studies have typically been performed with resting-state fMRI, and the functional connectivity is measured by the correlation of the BOLD signals between pairs of brain regions. A number of studies have provided evidence for different functional connectivities that may include the OFC, anterior cingulate cortex, amygdala and hippocampus, but many of these studies have involved relatively small numbers of participants, and whole-brain regions (Helm *et al.*, 2018). Indeed, power analyses show that resting-state functional connectivity for individual links in people is not robust with small samples (Button *et al.*, 2013; Poldrack, 2019), especially in populations with psychiatric disorders, as we also have found. The studies described next have therefore focused on large numbers of participants to provide robust results, and on voxel-level analysis to enable separation of connectivity of nearby brain areas such as the medial and lateral OFC. In addition, the studies considered next also have the advantage that in several it was possible to perform neuroimaging in a large sample of unmedicated patients and to compare the functional connectivity with that in medicated patients. Descriptions of some of the studies in which this has been possible are provided next.

### A brain-wide analysis of voxel-level differences in functional connectivity implicates the lateral and medial orbitofrontal cortex in depression

**Figure 7:**
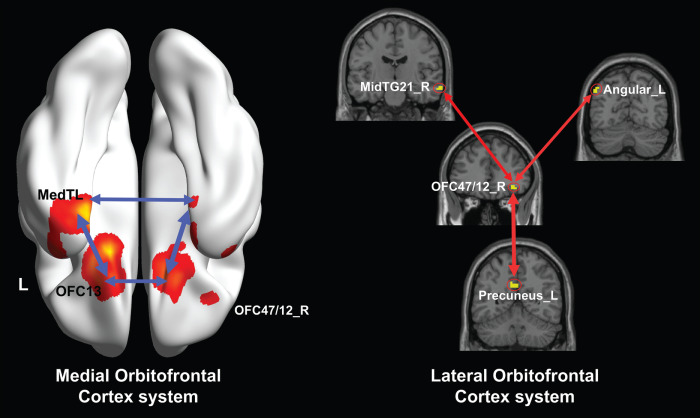
**Resting-state functional connectivity in depression.** The medial and lateral OFC networks that show different functional connectivity in patients with depression. A decrease in functional connectivity is shown by blue arrows, and an increase by red arrows. MedTL—medial temporal lobe from the parahippocampal gyrus to the temporal pole; MidTG21R—middle temporal gyrus area 21 right; OFC13—medial OFC area 13; OFC47/12R—lateral OFC area 47/12 right. The lateral OFC cluster in OFC47/12 is visible on the ventral view of the brain anterior and lateral to the OFC13 clusters (from Cheng *et al.*, 2016).

In the first brain-wide voxel-level resting-state functional connectivity neuroimaging analysis of depression (with 421 patients with major depressive disorder and 488 controls), we found that one major circuit with altered functional connectivity involved the medial OFC BA 13, which had reduced functional connectivity in depression with memory systems in the parahippocampal gyrus and medial temporal lobe (Cheng *et al.*, 2016) (Fig. 7). The lateral OFC BA 47/12, involved in non-reward and punishing events, did not have this reduced functional connectivity with memory systems, so that there is an imbalance in depression towards decreased reward-related connectivity with the medial temporal lobe memory system.

A second major circuit change was that the lateral OFC area BA 47/12 had increased functional connectivity with the precuneus, the angular gyrus and the temporal visual cortex BA 21 (Cheng *et al.*, 2016) (Fig. 7). This enhanced functional connectivity of the non-reward/punishment system (BA 47/12) with the precuneus [involved in the sense of self and agency (Rolls, 2021a)], and the angular gyrus [involved in language (Rolls, 2021a)] is thus related to the explicit affectively negative sense of the self, and of self-esteem, in depression.

The differences in orbitofrontal connectivity with these brain regions were related to the depression by evidence that the symptoms of depression were correlated with these differences of functional connectivity; and that the lateral OFC functional connectivity links described were less high if the patients were receiving antidepressant medication (Cheng *et al.*, 2016).

Because the lateral OFC responds to many punishing and non-rewarding stimuli that are likely to elicit autonomic/visceral responses via the anteroventral insula (see above), and in view of connections from these areas to the anterior insula which is implicated in autonomic/visceral function (Critchley and Harrison, 2013; Rolls, 2016c), the anterior insula would also be expected to be overactive in depression, which it is (Drevets, 2007; Hamilton *et al.*, 2013; Ma, 2015).

These advances were made possible because we performed whole-brain voxel-level functional connectivity, enabling clear separation and localization of differences between the lateral and medial OFC. Further analyses which focused instead on voxel-level functional connectivity of particular brain systems has revealed much more about the different systems involved, as described next, and have provided cross-validation in a cohort from the USA (Cheng *et al.*, 2018d).

### Precuneus: higher connectivity with the lateral orbitofrontal cortex

The precuneus is a medial parietal cortex region implicated in the sense of self and agency, autobiographical memory, spatial function. and navigation (Cavanna and Trimble, 2006; Freton *et al.*, 2014). The retrosplenial cortex provides connections and receives connections from the hippocampal system, connecting especially with the parahippocampal gyrus areas TF and TH, and with the subiculum (Kobayashi and Amaral, 2003, 2007; Bubb *et al.*, 2017). The precuneus provides access to the hippocampus for spatial information from the parietal cortex (given the rich connections between the precuneus and the parietal cortex) (Rolls and Wirth, 2018; Rolls, 2021a).

**Figure 8:**
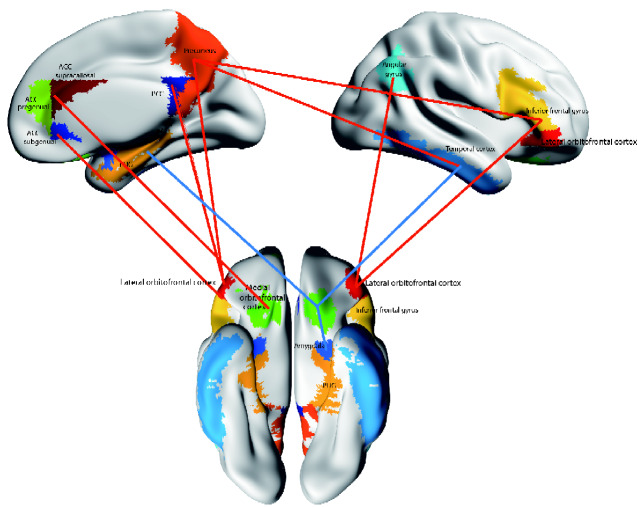
**Functional connectivity (FC) differences of the medial and lateral OFC in major depressive disorder.** Higher functional connectivity in depression is shown by red connecting lines, and includes higher functional connectivity of the non-reward/punishment-related lateral OFC with the precuneus, posterior cingulate cortex (PCC), pregenual anterior cingulate cortex (ACC), angular gyrus, and inferior frontal gyrus. Lower functional connectivity in depression is shown by blue connecting lines, and includes lower functional connectivity of the medial OFC with the parahippocampal gyrus memory system (PHG), amygdala, temporal cortex and supracallosal anterior cingulate cortex (ACC). The part of the medial OFC in which voxels were found with lower functional connectivity in depression is indicated in green. The areas apart from the medial OFC shown are as defined in the automated anatomical labelling atlas 2 (Rolls *et al.*, 2015a), although the investigations that form the basis for the summary were at the voxel level.

To further analyse the functioning of the precuneus in depression, resting-state functional connectivity was measured in 282 patients with major depressive disorder and 254 controls (Cheng *et al.*, 2018c). In 125 patients not receiving medication, voxels in the precuneus had significantly higher functional connectivity with the lateral OFC (Fig. 8). In patients receiving medication, the functional connectivity between the lateral OFC and the precuneus was decreased back towards that in the controls (Cheng *et al.*, 2018c). These findings support the theory that the non-reward system in the lateral OFC has increased effects on areas in which the self is represented including the precuneus, which could relate to the low self-esteem in depressed patients (Rolls, 2016a).

### Parahippocampal gyrus/medial temporal lobe memory system, and temporal lobe visual cortex: lower connectivity with the medial orbitofrontal cortex

We found that voxels in the medial OFC had lower functional connectivity with the parahippocampal gyrus/medial temporal lobe memory system (Cheng *et al.*, 2016) (Fig. 7), and interpreted this as resulting in fewer happy memories being recalled, as the medial OFC has activations that correlate with subjective pleasantness, as described above, and the parahippocampal gyrus is a pathway in the hippocampal episodic memory system (Kesner and Rolls, 2015; Rolls, 2016b, 2018b, 2019b, 2021a; Rolls and Wirth, 2018). The reduced connectivity with temporal cortex areas in which objects and faces are represented was interpreted as contributing to the reduced positive valuation of signals involved in emotion such as the sight of face expressions, and of people (Hasselmo *et al.*, 1989; Critchley *et al.*, 2000).

In a further analysis that investigated the effects of antidepressant medication (Rolls *et al.*, 2020a), medial OFC voxels had lower functional connectivity with temporal cortex areas, the parahippocampal gyrus, fusiform gyrus, and supplementary motor area, and medication did not result in these functional connectivities being closer to controls. This is consistent with the anhedonia of depression and reduced happy memories being related to these low functional connectivities of the medial OFC with temporal lobe and memory systems. What is especially interesting is that these low functional connectivities are not normalized by treatment with antidepressant drugs (Rolls *et al.*, 2020a), suggesting that one goal of future treatment for depression might be to increase the functionality of the medial OFC.

### Posterior cingulate cortex: higher functional connectivity with the lateral orbitofrontal cortex

The posterior cingulate cortex is a region with strong connectivity in primates with the entorhinal cortex and parahippocampal gyrus (areas TF and TH), and thus with the hippocampal memory system (Vogt, 2009; Bubb *et al.*, 2017; Rolls and Wirth, 2018; Rolls, 2018b; 2019b,c, 2021a). The posterior cingulate cortex also has connections with the OFC (Vogt and Pandya, 1987; Vogt and Laureys, 2009), and the posterior cingulate cortex has high functional connectivity with the parahippocampal regions that are involved in memory (Cheng *et al.*, 2018b; Rolls, 2019b). The posterior cingulate region (including the retrosplenial cortex) is consistently engaged by a range of tasks that examine episodic memory including autobiographical memory, and imagining the future; and also spatial navigation and scene processing (Auger and Maguire, 2013; Leech and Sharp, 2014). Self-reflection and self-imagery activate the ventral part of the posterior cingulate cortex (the part with which we will be mainly concerned here) (Kircher *et al.*, 2000, 2002; Johnson *et al.*, 2002; Sugiura *et al.*, 2005).

A study with 15 patients and 15 controls had implicated functional connectivity of the posterior cingulate with the subgenual cingulate cortex to rumination in depression (Berman *et al.*, 2011), but we find that functional connectivity in people with psychiatric disorders is not robust with small samples. Therefore, to analyse the functioning of the posterior cingulate cortex in depression, we performed a full voxel-level resting-state functional connectivity neuroimaging analysis of depression of the posterior cingulate cortex, with 336 patients with major depressive disorder and 350 controls (Cheng *et al.*, 2018b). In depression, the posterior cingulate cortex had significantly higher functional connectivity with the lateral OFC (Fig. 8). In patients receiving medication, the functional connectivity between the lateral OFC and the posterior cingulate cortex was decreased back towards that in the controls. These findings are consistent with the hypothesis that the non-reward system in the lateral OFC has increased effects on memory systems, which contribute to the rumination about sad memories and events in depression (Cheng *et al.*, 2018b).

### Anterior cingulate cortex: reduced connectivity with the orbitofrontal cortex in depression

The OFC projects to the anterior cingulate cortex (Vogt, 2009, 2019; Rolls, 2019b). The supracallosal anterior cingulate cortex is activated by many aversive stimuli (Grabenhorst and Rolls, 2011, Rolls, 2014, 2019b), and has strong connectivity with the lateral OFC and adjoining part of the inferior frontal gyrus (Rolls *et al.*, 2019; Hsu *et al.*, 2020; Du *et al.*, 2020a). The pregenual cingulate cortex is activated by many pleasant, rewarding, stimuli (Grabenhorst and Rolls, 2011; Rolls, 2014, 2019b), and has strong functional connectivity with the medial OFC (Rolls *et al.*, 2019; Hsu *et al.*, 2020; Du *et al.*, 2020a). However, the anterior cingulate cortex appears to be involved in learning actions to obtain rewards (action–outcome learning), where the outcome refers to the reward or punisher for which an action is being learned (Rudebeck *et al.*, 2008; Camille *et al.*, 2011; Rushworth *et al.*, 2011, 2012; Rolls, 2019b,c). In contrast, the medial OFC is involved in reward-related processing and learning, and the lateral OFC in non-reward and punishment-related processing and learning (Rolls, 2019c). These involve stimulus–stimulus associations, where the second stimulus is a reward (or its omission), or a punisher (Rolls, 2019c). Given that emotions can be considered as states elicited by rewarding and punishing stimuli, and that moods such as depression can arise from prolonged non-reward or punishment (Rolls, 2016a, 2018a, 2019c), the part of the brain that processes these stimulus–reward associations, the OFC, is more likely to be involved in depression than the action-related parts of the cingulate cortex. However, the action-related parts of the cingulate cortex, and other regions related to action such as the right inferior frontal gyrus, could contribute to the motor-related slowing, fatigue and decreased energy that are all symptoms commonly seen in depressed individuals (Rolls *et al.*, 2020a).

The subgenual (or subcallosal) cingulate cortex has been implicated in depression, and electrical stimulation in that region may relieve depression (Mayberg, 2003; Hamani *et al.*, 2009, 2011; Lozano *et al.*, 2012; Laxton *et al.*, 2013; Lujan *et al.*, 2013) [although it has not been possible to confirm this in a double-blind study (Holtzheimer *et al.*, 2017)]. However, the subgenual cingulate cortex is also implicated in autonomic function (Gabbott *et al.*, 2003), and this could be related to some of the effects found in this area that are related to depression. Whether the subgenual cingulate cortex is activated because of inputs from the OFC, or performs separate computations, is not yet clear. Furthermore, the possibility is considered that electrical stimulation of the subcallosal region, which includes parts of the ventromedial prefrontal cortex (Laxton *et al.*, 2013), that may relieve depression, may do so at least in part by activating connections involving the OFC, other parts of the anterior cingulate cortex, and the striatum (Johansen-Berg *et al.*, 2008; Hamani *et al.*, 2009; Lujan *et al.*, 2013).

In a study of depression it was found in unmedicated patients that the lateral orbitofrontal cortex where it becomes adjacent with the anteroventral insular cortex had increased functional connectivity with the subgenual / subcallosal anterior cingulate cortex (Fig. 8) (see Fig. S2B of (Rolls *et al.*, 2019)). This may reflect increased effects of unpleasant states represented in the lateral orbitofrontal cortex on autonomic output in which the anteroventral insula and subgenual cingulate cortex are implicated (Rolls, 2021). Increased functional connectivity was also found between the medial orbitofrontal cortex and a region including parts of the supracallosal anterior cingulate cortex (see Fig. S2A in Rolls *et al*., (2019) (Fig. 8)). This may reflect reward inputs reaching a supracallosal anterior cingulate cortex region typically involved in representing aversive stimuli.

### Inferior frontal gyrus: increased connectivity with the lateral orbitofrontal cortex in depression

The lateral OFC projects to the inferior frontal gyrus, and, very interestingly, higher functional connectivity was found in depression of voxels in the right inferior frontal gyrus with voxels in the lateral and medial OFC, cingulate cortex, inferior and middle temporal gyrus and temporal pole, the angular gyrus, precuneus, hippocampus and mid- and superior frontal gyrus (Rolls *et al.*, 2020a) (Fig. 8). In medicated patients, these functional connectivities of the inferior frontal gyrus were lower and towards those in controls.

The hypothesis was proposed that one way in which the OFC influences behaviour in depression is via the right inferior frontal gyrus, which projects in turn to premotor cortical areas (Du *et al.*, 2020a). Consistent with the consequent hypothesis that the inferior frontal gyrus route may allow non-reward signals to have a too great effect to inhibit behaviour in depression, lesions of the right inferior frontal gyrus impair stopping in the stop-signal task, and produce impulsiveness (Aron *et al.*, 2004, 2014). Also consistent with the hypothesis, successful stopping in the stop-signal task is associated with high activation of the inferior frontal gyrus and lateral OFC (Deng *et al.*, 2017).

### Amygdala: reduced connectivity with the orbitofrontal cortex in depression

The amygdala is involved in emotion, though as shown above it may be overshadowed in humans by the OFC (LeDoux and Pine, 2016; Rolls, 2019c). Some relatively small-scale studies had shown different functional connectivities of the amygdala in depression (Connolly *et al.*, 2017; Helm *et al.*, 2018). In a large-scale study of depression, amygdala voxels had decreased functional connectivity with the medial OFC (involved in reward); the lateral OFC (involved in non-reward and punishment); temporal lobe areas [involved in visual and auditory perception including face expression analysis (Perrett *et al.*, 1982; Leonard *et al.*, 1985; Rolls, 2011, 2012a)]; and the parahippocampal gyrus (involved in memory; Fig. 8) (Cheng *et al.*, 2018a). This disconnectivity of the amygdala may contribute to the depression.

### Sleep, depression and increased lateral orbitofrontal cortex connectivity

Sleep is frequently impaired in depression (Becker *et al.*, 2017). To advance understanding of the brain regions involved in sleep and depression, the relation between functional connectivity, depressive symptoms (the Adult Self-Report Depressive Problems scores) and poor sleep quality was measured in 1017 participants from the general population in the Human Connectome Project (Cheng *et al.*, 2018d). The brain areas with increased functional connectivity of these common links related to both sleep and depressive scores included the lateral OFC; the dorsolateral prefrontal cortex; the anterior and posterior cingulate cortex; the insula; the parahippocampal gyrus and hippocampus; the amygdala; the temporal cortex; and the precuneus. A mediation analysis showed that these functional connectivities in the brain contribute to the relation between depression and poor sleep quality.

Evidence was also found in this general population that the Depressive Problems scores were correlated with functional connectivities between areas that included the lateral OFC, cingulate cortex, precuneus, angular gyrus and temporal cortex (Cheng *et al.*, 2018d). Part of the importance of this is that it provides strong support for a role of the lateral OFC in depression in a general population in the USA in which a tendency to have depressive problems could be assessed. This cross-validation in a completely different population and in people not selected to have depression (Cheng *et al.*, 2018d) provides support for the theory that the lateral OFC is a key brain area that might be targeted in the search for treatments for depression (Rolls, 2016a). Low sleep duration and depression are also related to structural differences of the OFC (Cheng *et al.*, 2020). In particular, higher depressive problems’ scores were associated with reduced cortical areas or volumes of brain regions that included the lateral and medial OFC, temporal cortex, precuneus, superior and middle frontal gyrus and superior medial frontal cortex, angular and supramarginal gyrus, and hippocampus (Cheng *et al.*, 2020).

### Effective connectivity in depression

Effective connectivity measures the effect of one brain region on another in a particular direction, and can, in principle, therefore provide information related to the causal processes that operate in brain function, that is, how one brain region influences another.

In a resting-state fMRI investigation, effective connectivity directed to the medial OFC from areas including the parahippocampal gyrus, temporal pole, inferior temporal gyrus and amygdala was decreased in depression (Rolls *et al.*, 2018). This implies less strong positive driving influences of these input regions on the medial and middle OFC, regions implicated in reward, and thus helps to elucidate part of the decreased feelings of happy states in depression (Rolls, 2016a). The links from temporal cortical areas to the precuneus were increased in depression, and this may relate to representations of the sense of self (Cavanna and Trimble, 2006), which become more negative in depression (Cheng *et al.*, 2016; Rolls, 2016a). The lateral OFC, implicated in non-reward and punishment, had an increased level of activity as reflected in the model in the depressed group. In addition, activity in the model was also higher in the right and left hippocampus of patients with depression, implying heightened memory-related processing (Rolls *et al.*, 2018).

## Orbitofrontal cortex activations to reward and non-reward in depression

### Reinforcement learning approaches

Depression has been investigated in the framework of reinforcement learning, using in particular the learning rate coefficient and the sensitivity to reward. In earlier studies, it was reported that depression reduces prediction errors during reinforcement learning (Kumar *et al.*, 2008; Gradin *et al.*, 2011), but Rutledge *et al.* (2017) found that prediction error was unchanged in the ventral striatum. On that basis, they suggested that depression does not affect the expression of dopaminergic reward prediction errors. Their study was consistent with evidence that reward sensitivity and not learning rates are reduced in anhedonic depression (Huys *et al.*, 2013; Chen *et al.*, 2015). Also within the reinforcement learning framework, it was found that positive reward prediction error in the medial OFC is reduced in depression, and was correlated with anhedonia, but the learning was intact, in 28 drug-naive patients with depression (Rothkirch *et al.*, 2017).

### Increased activations to non-reward of the lateral orbitofrontal cortex, and decreased sensitivity to reward of the medial orbitofrontal cortex are related to depression scores

In 1140 adolescents at age 19 and 1877 at age 14 in the monetary incentive delay task, the medial OFC had graded increases in activation as the reward (Win) value increased (Xie *et al.*, 2020). The lateral OFC had graded increases of activation as the reward value dropped to zero (the No-Win condition) (Fig. 5).

In a subgroup with a high score on the Adolescent Depression Rating Scale at the age of 19 and 14, the medial OFC activations had reduced sensitivity to the different reward conditions; and the lateral OFC activation showed high activation to the No-Win (i.e. Non-reward) condition (Xie *et al.*, 2020). These new findings provide support for the hypothesis that those with symptoms of depression have increased sensitivity to non-reward in the lateral OFC, and decreased sensitivity for differences in reward of the medial OFC. Moreover, these differences are evident at an age as early as 14 years old (Xie *et al.*, 2020). This result thus supports the theory that depressive symptoms can be related to sensitivity to non-reward (Rolls, 2016a, 2018a), that is, to not winning in this monetary reward task.

## Possible structural differences in the orbitofrontal cortex in depression

For completeness, we note that there is some evidence for altered structure and function of the lateral OFC in depression (Drevets, 2007; Price and Drevets, 2012; Ma, 2015). For example, reductions of grey-matter volume have been demonstrated specifically in the posterolateral OFC (BA 47, caudal BA 11 and the adjoining BA 45), and also in the subgenual cingulate cortex (BA 24, 25) (Nugent *et al.*, 2006; Drevets, 2007; Grieve *et al.*, 2013). Meta-analyses revealed that depressed patients showed large volume reductions in frontal regions, especially in the anterior cingulate and OFC (Koolschijn *et al.*, 2009; Lorenzetti *et al.*, 2009).

In recent large-scale studies with the Adolescent Brain Cognitive Developmental (ABCD) data set, it has been found that a number of factors are associated with psychiatric problems including the depressive problems score, and with reduced brain volume. The brain regions with reduced volume include the OFC, hippocampus, temporal cortex and medial frontal cortex. The factors that are associated with these differences are low maternal age (Du *et al.*, 2020b), problems in the family (Gong *et al.*, 2020), severe nausea and vomiting in pregnancy (Wang *et al.*, 2020) and low sleep duration (Cheng *et al.*, 2020).

In depression, there is increased cerebral blood flow in areas that include the ventrolateral OFC (which is a prediction of the theory), and also in regions such as the subgenual cingulate cortex and amygdala, and these increases appear to be related to the mood change, in that they become more normal when the mood state remits (Drevets, 2007), but convergence across studies is not strong (Gray *et al.*, 2020).

## The orbitofrontal cortex and possible treatments for depression, including new areas for brain stimulation

In research stimulated by the theory (Rolls, 2016a), it has been reported that transcranial magnetic stimulation of the right lateral OFC, which may disrupt its activity, helps in the treatment of depression in a substantial proportion of patients (Feffer *et al.*, 2018; Downar, 2019).

Treatment with antidepressant drugs decreases the activity (Ma, 2015) and functional connectivity (Cheng *et al.*, 2016, 2018b,c; Rolls *et al.*, 2019, 2020a) of the non-reward lateral OFC system. The research described suggests that a search for new treatments that would increase the connectivity of the reward-related medial OFC could be helpful because current medications do not ameliorate these reduced functional connectivities.

Deep brain stimulation of the OFC may also be useful in the treatment of mood disorders and depression. The macaque OFC is a key brain site at which deep brain electrical stimulation is rewarding (Rolls *et al.*, 1980; Rolls, 2005, 2019c). Electrical stimulation of the human OFC can also produce reward and raise mood (Rao *et al.*, 2018), and many of the sites were in the middle part of the OFC, areas 13 and 11, which are categorized as medial OFC, the area activated by rewards (Rolls, 2019c). It is likely that these medial OFC sites will produce better reward in humans than stimulation in the lateral OFC BA12/47, for these lateral sites are activated by unpleasant stimuli and by not obtaining expected rewards. The medial (/middle) OFC may, for the reasons described here and elsewhere (Rolls, 2019c), be a key area of interest for deep brain stimulation to help relieve depression.

The anterior cingulate cortex, including the subcallosal cingulate cortex, is a key brain region to which the OFC projects (Rolls, 2019b). It is possible that brain stimulation of the subcallosal cingulate cortex might be useful in the treatment of at least some patients with depression (Johansen-Berg *et al.*, 2008; Lujan *et al.*, 2013; Dunlop *et al.*, 2017; Holtzheimer *et al.*, 2017; Riva-Posse *et al.*, 2018), and it is possible that the subcallosal cingulate stimulation affects pathways that connect with the OFC (Johansen-Berg *et al.*, 2008; Lujan *et al.*, 2013; Dunlop *et al.*, 2017; Riva-Posse *et al.*, 2018). Given that the anterior cingulate cortex is an output region of the OFC (Fig. 1), it may be that treatments of the OFC, where the emotion is implemented, may be a better target for potential treatments for depression.

The general approach to depression described here, that it relates to effects produced by increased non-reward or non-reward sensitivity, or decreased reward or reward sensitivity (Fig. 6), has implications for self-help and behavioural treatments for depression (Rolls, 2018a), as well as for medical interventions.

## What are the special computational roles of the orbitofrontal cortex in reward, emotion and decision-making?

It is important in terms of our understanding of brain function to consider what is special about the primate including human OFC for reward, decision-making and emotion, compared to other brain regions. This invites an answer about what is special about the computations performed by the OFC compared to other brain regions (Rolls, 2016b, 2019c, 2021a).

First, the OFC, as a neocortical area, has highly developed recurrent collateral connections between its pyramidal cells, which together with associative synaptic plasticity, provide the basis for auto-association or attractor networks (Rolls, 2016b, 2021a). [The amygdala, in contrast, has little recurrent collateral connectivity (Millhouse and DeOlmos, 1983).]

These attractor networks provide the basis for short-term memory functions, by maintaining neuronal firing in a stable attractor (Rolls, 2021a). These attractor networks can hold on-line which stimuli (and this could be other individuals) are currently rewarding, which is important for social interactions and economic decisions. This memory capability is an important component of rule-based one-trial reversal, in which the current rule must be held in short-term memory (Deco and Rolls, 2005). Short-term memory is also potentially very useful for holding mood online for some time, so that if, for example, a reward is not received, the non-reward state of frustration can lead to continuing attempts to regain the reward. Similar short-term memory processes might enable one to remember the recent reinforcement history of individuals, and again can be important in decision-making. The short-term memory aspects of these attractor networks are also important for holding the expected value online, until the reward outcome is received, after which non-reward neurons may be activated in ways for which there is a computational model (Rolls and Deco, 2016), and such computations may also contribute to reward prediction error, defined as the reward outcome value minus the expected reward. The short-term memory also provides the biasing system for top-down attention to reward value (Grabenhorst and Rolls, 2008, 2010; Rolls *et al.*, 2008; Ge *et al.*, 2012; Luo *et al.*, 2013), and attention by biased activation (Rolls, 2013a, 2021a).

These attractor networks in the OFC and ventromedial prefrontal cortex also provide the basis for reward-related decision-making, in which inputs to two competing attractor states in an attractor network lead to a bifurcation and to a decision (Rolls and Deco, 2010; Rolls *et al.*, 2010a,b, Deco *et al.*, 2013; Rolls, 2021a). Part of the utility of this approach to decision-making is that once the decision has been taken in the attractor network, the results of the decision are kept active in the decision-making attractor network to provide the goals for the selection of actions by the cingulate cortex to obtain the rewards (Rolls, 2019b,c, 2021a).

Second, the primate and human OFC as a neocortical area is beautifully connected anatomically to receive inputs from representations of ‘what’ stimulus is present from every sensory modality at the top of each sensory cortical hierarchy, independently of reward value (Fig. 2), and then to compute multimodal representations that are then represented in terms of their reward value. This is very different from the rodent, in which reward is represented throughout the processing systems (Rolls, 2019c, 2021a).

Third, the primate OFC specializes in reward value, rather than action. This separation allows the value of many stimuli in the high dimensional space of different rewards (Rolls, 2014) to be represented, and for competition between them to be useful for computing relative reward value (Grabenhorst and Rolls, 2009). Moreover, mood states can be maintained, independently of any actions being performed. In contrast, as described above, the rodent ‘OFC’ is also involved in motor responses and actions (Wilson *et al.*, 2014), so it can be less specialized for representing reward value, and rapidly changing it.

Fourth, the primate OFC projects reward value representations to the pregenual anterior cingulate cortex, and punishment value representations to the supracallosal anterior cingulate cortex (Hsu *et al.*, 2020; Du *et al.*, 2020a), where they can be associated with actions performed recently, received via the posterior cingulate cortex from the parietal cortex, to implement action–outcome learning. Outputs are then directed from mid-cingulate areas to the premotor cortical areas (Rolls, 2019b). The amygdala does not have similar connectivity.

Fifth, the human lateral OFC has considerable connectivity with the inferior frontal gyrus areas 45 and 44 which in the left are Broca’s area, and this may be part of the route by which the OFC, especially laterally, becomes functionally connected with language areas in the posterior temporal and parietal areas (Hsu *et al.*, 2020; Du *et al.*, 2020a; Rolls, 2021a). This provides a route for top-down influences of language-related processing on emotional and social behaviour, and indeed it is part of the long-loop interactions between attractor networks that are proposed to contribute to increased rumination in depression (Cheng *et al.*, 2016; Rolls, 2016a, 2021a).

## Conclusions

The research described here provides evidence that the primate including human OFC is a key brain region in reward value representation and in emotion (Rolls, 2019c). The primate OFC represents values, but not actions. Instead, the OFC sends reward outcome information to the cingulate cortex for use in action–outcome goal-directed learning, both of which are therefore key to understanding emotions and goal-directed actions (Rolls, 2019b). The case is also made that the OFC is a source of reward-related information that reaches the dopamine neurons in the brainstem (Rolls, 2017; Namboodiri *et al.*, 2019) (Fig. 2), which provide for reinforcement-based learning of stimulus–response habits in the basal ganglia.

This framework provides a foundation for the proposal that because of its importance in emotion, the OFC is likely to be a key brain region in emotional disorders such as depression. The new evidence from large-scale voxel-level neuroimaging studies described here shows that the reward-related medial OFC has reduced functional connectivity with a number of brain systems including the medial temporal lobe memory system. Interestingly, antidepressant medications do not normalize these functional connectivities, suggesting a new avenue for exploration for new treatments for depression. The new evidence also shows that the punishment/non-reward-related lateral OFC and its related nearby right inferior frontal gyrus areas have increased functional connectivity in depression, which may relate to increased non-reward processing in depression. These advances, and the way in which depression can be related to altered responsiveness to non-rewards, provide an approach to the better understanding and treatment of depression (Rolls, 2016a, 2018a, 2019c, 2021a). Moreover, these new approaches relate to ideas that the subgenual cingulate cortex is involved in depression, for it receives major inputs from the OFC.

The research described here provides a theory supported by much empirical evidence for why and how the OFC is involved in depression, and provides a complementary approach to the strong focus there has been on the subgenual or subcommissural cingulate cortex in depression (Mayberg, 2003; Hamani *et al.*, 2011; Lozano *et al.*, 2012; Mayberg *et al.*, 2016; Dunlop *et al.*, 2017; Riva-Posse *et al.*, 2018). We hope that the new concepts and evidence presented here based on a fundamental understanding of the functions of the OFC in emotion will help in developments of better understanding and treatments for depression (Rolls, 2018a).

## Technical note

The orbitofrontal cortex is a difficult brain region for fMRI as sometimes signal dropout and distortion can occur due to inhomogeneities in the magnetic field being produced by the air-filled bony sinuses. For many of our investigations, we utilized a protocol set-up very helpfully by Prof Peter Jezzard (FMRIB, Oxford) for imaging in the coronal plane with the head angle optimized for each participant, and with imaging parameters that were carefully selected, as described in our articles before 2012. Since then, we have successfully used imaging in approximately the plane of the Sylvian fissure, which enables imaging of both the OFC and the medial temporal lobe, as set out in the previously published articles (Deichmann *et al.*, 2002, 2003; Rolls *et al.*, 2015b, 2020b).

## Funding

This work was supported as follows: J. F. was supported by National Key R&D Program of China (No. 2019YFA0709502 and 2018YFC1312904); the 111 Project (No. B18015); Shanghai Municipal Science and Technology Major Project (No. 2018SHZDZX01); ZJLab; Shanghai Center for Brain Science and Brain-Inspired Technology; and National Key R&D Program of China (No 2018YFC1312900). W.C. was supported by grants from the National Natural Sciences Foundation of China (No. 82071997 and 81701773) and Natural Science Foundation of Shanghai (No. 18ZR1404400).

## Conflict of interest

The authors report no competing interests.

## Data availability

Data sharing is not applicable to this article as no new data were created or analysed in this study.

## Abbreviations

ADHD =: attention deficit hyperactivity disorder
BA =: Brodmann area
BOLD =: blood oxygenation-level dependent
fMRI =: functional magnetic resonance imaging
OFC =: orbitofrontal cortex
VLPFC =: ventrolateral prefrontal cortex
VMPFC =: ventromedial prefrontal cortex
